# Intestine-Specific Mttp Deletion Decreases Mortality and Prevents Sepsis-Induced Intestinal Injury in a Murine Model of *Pseudomonas aeruginosa* Pneumonia

**DOI:** 10.1371/journal.pone.0049159

**Published:** 2012-11-08

**Authors:** Jessica A. Dominguez, Yan Xie, W. Michael Dunne, Benyam P. Yoseph, Eileen M. Burd, Craig M. Coopersmith, Nicholas O. Davidson

**Affiliations:** 1 Department of Surgery, Washington University School of Medicine, St. Louis, Missouri, United States of America; 2 Department of Medicine, Washington University School of Medicine, St. Louis, Missouri, United States of America; 3 Department of Pathology & Immunology, Washington University School of Medicine, St. Louis, Missouri, United States of America; 4 Emory Center for Critical Care and Department of Surgery, Emory University School of Medicine, Atlanta, Georgia, United States of America; 5 Department of Pathology and Laboratory Medicine, Emory University School of Medicine, Atlanta, Georgia, United States of America; Charité, Campus Benjamin Franklin, Germany

## Abstract

**Background:**

The small intestine plays a crucial role in the pathophysiology of sepsis and has been referred to as the “motor” of the systemic inflammatory response. One proposed mechanism is that toxic gut-derived lipid factors, transported in mesenteric lymph, induce systemic injury and distant organ failure. However, the pathways involved are yet to be defined and the role of intestinal chylomicron assembly and secretion in transporting these lipid factors is unknown. Here we studied the outcome of sepsis in mice with conditional, intestine-specific deletion of microsomal triglyceride transfer protein (*Mttp-IKO*), which exhibit a block in chylomicron assembly together with lipid malabsorption.

**Methodology/Principal Findings:**

*Mttp-IKO* mice and controls underwent intratracheal injection with either *Pseudomonas aeruginosa* or sterile saline. *Mttp-IKO* mice exhibited decreased seven-day mortality, with 0/20 (0%) dying compared to 5/17 (29%) control mice (p<0.05). This survival advantage in *Mttp-IKO* mice, however, was not associated with improvements in pulmonary bacterial clearance or neutrophil infiltration. Rather, *Mttp-IKO* mice exhibited protection against sepsis-associated decreases in villus length and intestinal proliferation and were also protected against increased intestinal apoptosis, both central features in control septic mice. Serum IL-6 levels, a major predictor of mortality in human and mouse models of sepsis, were elevated 8-fold in septic control mice but remained unaltered in septic *Mttp-IKO* mice. Serum high density lipoprotein (HDL) levels were reduced in septic control mice but were increased in septic *Mttp-IKO* mice. The decreased levels of HDL were associated with decreased hepatic expression of apolipoprotein A1 in septic control mice.

**Conclusions/Significance:**

These studies suggest that strategies directed at blocking intestinal chylomicron secretion may attenuate the progression and improve the outcome of sepsis through effects mediated by metabolic and physiological adaptations in both intestinal and hepatic lipid flux.

## Introduction

Pneumonia is an important cause of mortality in developed countries and is implicated as a cause of death in 50,000 patients a year in the United States. Patients who contract pneumonia often succumb despite aggressive treatment with targeted antimicrobial therapies [1]. Among the complications of pneumonia, patients develop multi-organ dysfunction syndrome and mortality, at least in part, from the extrapulmonary complications and frequently meet diagnostic criteria for sepsis and the systemic inflammatory response syndrome (SIRS) [2]. Despite the importance of early intervention with antibiotic therapy, there is a pressing and currently unmet need to validate additional strategies to mitigate the effects of systemic sepsis [3].

Emerging information suggests that the small intestine plays a central role in the pathophysiology of sepsis and has been referred to as the “motor” of SIRS [4], [5]. Among the proposed mechanisms, one suggested pathway is that toxic gut-derived lipid factors, transported in the mesenteric lymph, secondarily induce systemic injury and distant organ failure [6], [7]. Other evidence in support of this general “gut-lymph hypothesis” is that lung injury induced by trauma-hemorrhagic shock can be abrogated by ligation of the mesenteric lymph duct [6]–[9]. These findings collectively point to the transport of gut-derived factors in mediating systemic effects from distant (non-intestinal) injury. In relation to the role of dietary modulation of intestinal lipid metabolism, studies have demonstrated that mice fed a high fat diet for several weeks exhibit impaired ability to clear *Staphylococcus aureus* bacteremia following intravenous injection [10] while yet other studies demonstrated that short term high fat feeding led to increased mortality and end organ injury following cecal ligation and puncture [11]. Taken together, the published evidence strongly suggests that alterations in intestinal lipid metabolism may influence the host response to infection. However, the precise pathways involved and in particular the role of chylomicron assembly and secretion are poorly understood.

Systemic sepsis has also been shown to influence intestinal epithelial turnover kinetics and functional parameters including increasing the rate of epithelial apoptosis [12]–[15], decreasing proliferation [16], [17], altering the production of cytokines [18], and also inducing intestinal barrier dysfunction [19]–[21]. Many of these perturbations have been identified in models of pneumonia-induced sepsis. In addition, prevention or attenuation of some of these manifestations of sepsis-induced gut epithelial apoptosis is associated with increased survival [15].

In the current study we have evaluated the role of intestinal chylomicron assembly and secretion in order to understand the pathways involved in the transport of gut-derived lipid factors in the setting of sepsis. For this purpose, we used a line of mice with defective chylomicron assembly which is induced following conditional, intestine-specific deletion of microsomal triglyceride transfer protein (*Mttp-IKO*) [22]. Mttp is a lipid transfer protein that resides within the lumen of the endoplasmic reticulum and plays a requisite role in the assembly of triglyceride-rich lipoprotein particles, both within enterocytes of the small intestine (ie chylomicrons) and also in hepatocytes of the liver (ie very low density lipoproteins, VLDL) [23]. Animals of the indicated genotype were subjected to experimentally induced *Pseudomonas aeruginosa* pneumonia, the most common cause of gram negative nosocomial pneumonia [24]. We find that *Mttp-IKO* mice exhibit a survival advantage in association with intestinal adaptation including attenuation of the sepsis-associated changes in villus length, intestinal proliferation and apoptosis and in conjunction with altered local and systemic cytokines profiles.

## Materials and Methods

### Animals

Mttp^flox/flox^ villin-Cre-ER^T2^ (*Mttp-IKO*) mice in a background of ∼75% C57BL/6 and ∼25% 129/SvJ were used for these studies. Cre recombinase expression in villus epithelial cells was induced by five daily intraperitoneal injections of 1 mg tamoxifen (Sigma), as described previously [22]. Experiments were performed on mice between 8–10 weeks of age, and were undertaken 3 weeks after the tamoxifen injections, while animals were consuming regular low fat rodent chow. In all experiments, comparisons were made between 1) control sham and control septic mice, 2) *Mttp-IKO* sham and *Mttp-IKO* septic mice, 3) control sham and *Mttp-IKO* sham mice, and 4) control septic and *Mttp-IKO* septic mice. All animals were euthanized 24 h post-operatively or were followed for survival for 7 days, as indicated in the figure legends. A separate group of 24 C57BL/6 mice were fed either regular low fat rodent chow or a high fat diet (60% by calories) used to produce diet-induced obesity (Cat # D58Y1, TestDiet, Richmond, IN) for 3 weeks prior to pneumonia induction (described below). All animal studies were approved by the Animal Studies Committees of Washington University School of Medicine and Emory University School of Medicine and were conducted in accordance with the National Institutes of Health guidelines for the use of laboratory animals.

### Pneumonia Model

Pneumonia was induced by direct intratracheal instillation of *P. aeruginosa* (ATCC 27853) via a 29-gauge syringe under isoflurane anesthesia [17], [25]. This well characterized strain utilizes a type III secretion system and is *exoS* positive/*exoU* negative [26]. A total of 40 µl of bacteria diluted in normal saline was used, corresponding to 4×10^6^ colony-forming units. To enhance delivery of the bacteria into the lungs, mice were held vertically for 10 seconds. Sham mice were treated identically except they received intratracheal instillation of an equivalent volume of saline. All mice received a subcutaneous injection of 1 ml saline post-operatively to compensate for insensible fluid losses.

### Morphological Analysis of Intestine

Intestinal sections embedded in paraffin were stained with hematoxylin and eosin (H&E) for morphological analysis. Villus length and crypt depth were measured using Nikon Elements imaging software (Nikon Instruments, Melville, NY). Twelve well-oriented villi and crypts from each section were measured. Osmium tetroxide staining of intracellular lipid droplets was conducted using intestinal tissue fixed in 10% neutral buffered formalin, which was transferred into 1% osmium tetroxide with periodic shaking. The tissue was rinsed in distilled water and incubated in 0.5% periodic acid, washed and then processed for paraffin embedding and counterstaining with H&E.

### Apoptosis Quantification

Apoptotic cells in the proximal jejunum were quantified using two independent but complimentary techniques: active caspase-3 staining and morphologic analysis of H&E-stained sections [27]. Sections were deparaffinized, rehydrated, and incubated in 3% hydrogen peroxide for 10 minutes. Slides were then placed in Antigen Decloaker (Biocare Medical) and heated in a pressure cooker for 45 minutes, blocked with 20% normal goat serum (Vector Laboratories, Burlingame, CA), and incubated with rabbit polyclonal anti-active caspase-3 (1∶100; Cell Signaling, Beverly, MA) overnight at 4°C. Sections were then incubated with goat anti-rabbit biotinylated secondary antibody (1∶200; Vector Laboratories) for 30 minutes at room temperature followed by Vectastain Elite ABC reagent (Vector Laboratories) for 30 minutes at room temperature. Sections were developed with diaminobenzidine and counterstained with hematoxylin. For H&E-stained sections, apoptotic cells were identified using morphological criteria of cell shrinkage with condensed and fragmented nuclei. Apoptotic crypt epithelial cells were quantified in 100 well-oriented contiguous crypt-villus units.

### Real-time Quantitative Polymerase Chain Reaction and Western Blotting

Total RNA was isolated from frozen jejunal tissue using the RNeasy Mini Kit (QIAGEN, Santa Clarita, CA) or TRIzolP®^P^ Reagent (Invitrogen Life Technologies, Carlsbad, CA) according to the manufacturer’s protocol. Integrity of the RNA was verified by electrophoresis and cDNA was synthesized from 0.5 µg of total RNA. Bax, Bcl-2, and Bcl-xL mRNA levels were detected using pre-developed TaqMan primers and probes (Applied Biosystems, Foster, CA) and run on the ABI 7900HT Sequence Detection System (Applied Biosystems). Samples were run in duplicate and normalized to expression of the endogenous control, glyceraldehyde-3-phosphate (Applied Biosystems). Relative quantification of PCR products were based upon the value differences between the target gene and glyceraldehyde-3-phosphate using the comparative CT method. For expression of genes related to intestinal cholesterol efflux, qRT-PCR assays were performed in triplicate on an ABI Prism7000 sequence detection system using SYBR Green PCR Master Mix (Applied Biosystems) and primer pairs designed by Primer Express software (Applied Biosystems). Relative mRNA abundance is expressed as fold change compared to mRNA levels in control-sham mice, normalized to Gapdh. Western blotting was conducted using tissue lysates (100 mg) prepared in buffer containing 20 mM Tris, 1 mM sodium vanadate, 150 mM sodium chloride, 2 mM EDTA, 100 mM sodium fluoride, 50 mM β-glycerol-phosphate, 5% glycerol,1% TritonX100 containing. Complete protease inhibitor cocktail (Roche, NJ) and electrophoretic resolution by SDS-PAGE and transfer onto Immobilon membranes (Millipore, Billerica, MA). Western blots were conducted using antibodies to Abca1 (1∶1000, Novus Biologicals, CO), apolipoprotein A1 (1∶500) and A4 (1∶1000) previously generated in our laboratory [28].

### Intestinal Proliferation

Ninety minutes prior to euthanasia, mice were injected intraperitoneally with 5-Bromo-2′deoxyuridine (BrdU) (200 µl volume, 5 mg/ml diluted in normal saline; Sigma) to label cells in S-phase. Intestinal sections were then deparaffinized, rehydrated, and incubated in 1% hydrogen peroxide for 15 minutes. Slides were immersed in Antigen Decloaker (Biocare Medical, Concord, CA) and heated in a pressure cooker for 45 minutes, blocked with Protein Block (Dako, Carpinteria, CA) for 10 minutes, and incubated with rat monoclonal anti-BrdU overnight at 4°C (1∶500; Accurate Chemical & Scientific, Westbury, NJ). Sections were then incubated with goat anti-rat secondary antibody (1∶500; Accurate Chemical & Scientific) for 30 minutes at room temperature followed by streptavidin-horseradish peroxidase (1∶500; Dako) for 1 hour at room temperature. Slides were developed with diaminobenzidine, and counterstained with hematoxylin. BrdU-stained cells were quantified in 100 well-oriented contiguous crypts.

### Bacterial Cultures

Bronchoalveolar lavage (BAL) fluid was obtained following tracheal instillation with 1 ml sterile saline. BAL samples were serially diluted in sterile saline and plated on sheep’s blood agar plates. Plates were incubated overnight at 37°C in 5% CO2 and colony counts were determined 24 hours later. Colony counts were expressed as colony forming units (CFU)/ml of fluid and then converted to a logarithmic scale for statistical analysis.

### Myeloperoxidase (MPO) Activity

MPO activity was evaluated in BAL fluid to assess neutrophil infiltration. BAL fluid was collected as described above and centrifuged at 5,000 rpm for 5 minutes. Following addition of substrate buffer containing O-dianisidine and 0.0005% hydrogen peroxide, MPO activity was measured at 460 nm wavelength over 6 minutes (Bio-Tek Instruments-µQuant Microplate Spectrophotometer, Winooski, VT). MPO activity was calculated as optical density/minute (U) per µl of BAL fluid.

### Cytokine and Lipopolysaccharide (LPS) Levels

At 24 hr, blood was collected and serum obtained by centrifugation at 5,000 rpm for 5 minutes in serum separator tubes and stored at −80°C until use. Serum and BAL cytokine levels of IL-1β, IL-6, IL-10, IL-13, G-CSF, and TNF-α were measured by using a multiplex cytokine assay (Bio-Rad) according to manufacturer’s instructions. All samples were run in duplicate. For LPS determinations, sera were diluted 1∶10 and heated at 70°C for 15 minutes to inactive inhibitors. LPS was then measured using the LAL chromogenic endotoxin quantitation kit according the manufacturer’s instructions (ThermoScientific, Rockford, IL).

### Serum and Tissue Lipids

Blood was collected by retroorbital bleed immediately before surgery (pre-operative) and 24 hr after surgery (post-operative). Determinations of serum trigylcerides (TG), cholesterol, free fatty acids (FFA), and phospholipids (PL) were performed using kits obtained from Wako Chemicals (Richmond, VA). Lipid quantitation was also undertaken on the proximal jejunum of the indicated groups of mice. Fast protein liquid chromatography (FPLC) was undertaken as previously described to quantify HDL and LDL using tandem Superose 6 columns [22], [28].

### Statistical Analysis

Continuous data sets were tested for Gaussian distribution by using a normality test. Depending on this distribution, multiple group comparisons were performed either with one-way analysis of variance followed by the Newman-Keuls post-test or by the Kruskal-Wallis nonparametric one-way analysis of variance by ranks followed by the Dunn’s post-test. Survival studies were analyzed via the log-rank test. Data were analyzed using Prism 4.0 (GraphPad Software, San Diego, CA) and reported as means ± SEM. A p value <0.05 was considered to be statistically significant.

## Results

### Impaired Lipid Transport Confers a Survival Advantage in *P. aeruginosa* Pneumonia

**Figure 1:**
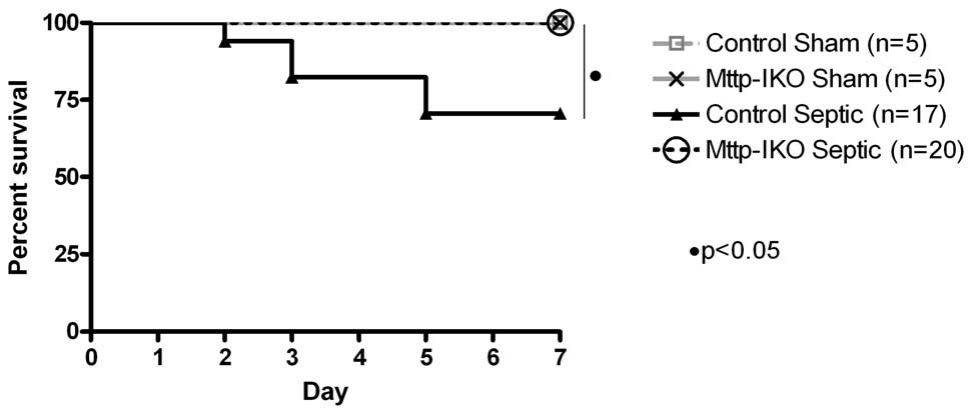
Effect of impaired intestinal lipid transport on mortality in *P. aeruginosa* pneumonia. Mice with intestine-specific deletion of microsomal triglyceride transfer protein (*Mttp-IKO*) and control mice were subjected to *P. aeruginosa* pneumonia. Sham mice received intratracheal injections of saline. All mice were followed for survival for 7 days. Mice with impaired intestinal lipid transport exhibited significantly improved survival compared to control mice (p<0.05). All sham mice survived.

Using the approaches outlined above, in which control and *Mttp-IKO* mice were subjected to *P. aeruginosa* pneumonia, we first established that impairment of chylomicron assembly confers a survival advantage in sepsis (Figure 1). Control mice with pneumonia exhibited a 29% seven-day mortality (5/17 mice), while *Mttp-IKO* mice with pneumonia exhibited 100% survival (20/20 mice) at seven days. All sham mice survived their operation.

### Pneumonia-induced Villus and Crypt Atrophy is Prevented in Mice with Impaired Lipid Transport

**Figure 2:**
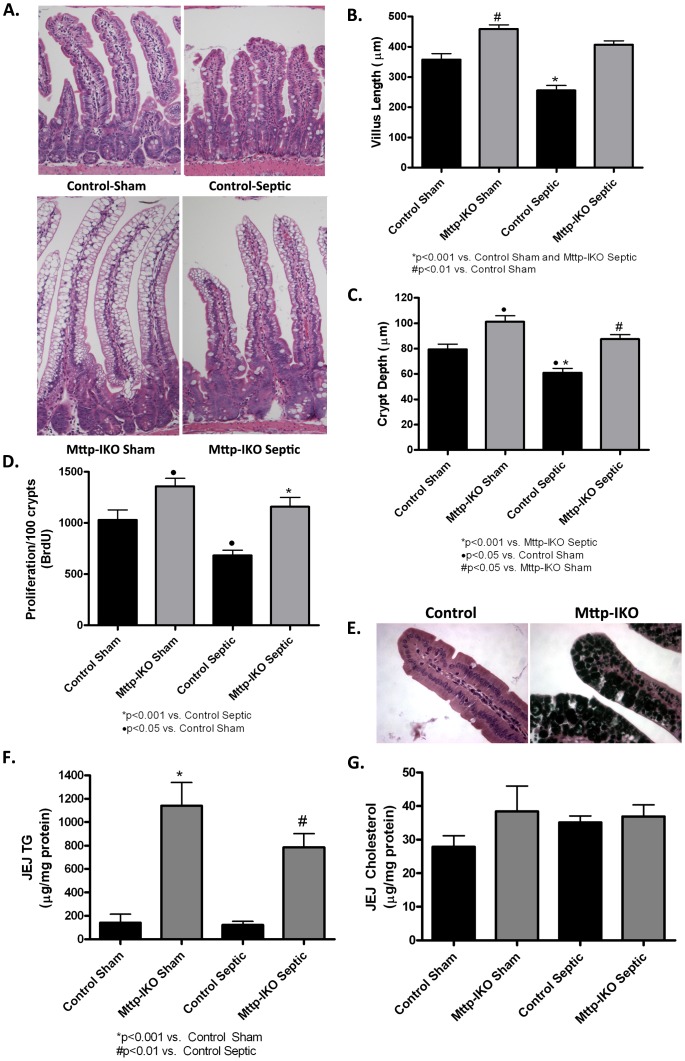
Effect of impaired intestinal lipid transport on intestinal morphology and proliferation. Intestinal morphology (A) was evaluated in H&E-stained sections. Magnification 20×. Villus length (B) and crypt depth (C) were quantified in jejunal sections. Control mice subjected to *P. aeruginosa* pneumonia had significantly shorter villi and smaller crypts than sham mice, while both villus length and crypt depth was restored or nearly restored to sham levels in septic *Mttp-IKO* mice. n = 9 shams/genotype, n = 17 septics/genotype. (D) S-phase cells were quantified in 100 crypts. Control mice subjected to *P. aeruginosa* pneumonia had significantly decreased intestinal proliferation compared to control sham mice, while septic *Mttp-IKO* mice exhibited increased proliferative capacity. n = 6–7 shams/genotype, n = 9–11 septics/genotype. (E) Intestinal tissue was stained with osmium to detect intracellular lipid droplets, which appear as dark black staining material. (F) Mucosal concentrations of triglycerides and (G) cholesterol were measured in jejunum, the data expressed as µg/mg protein. n = 5/group.

Mice with pneumonia and sepsis exhibited a decrease in villus length and crypt depth, changes that were markedly attenuated in septic *Mttp-IKO* mice (Figure 2A–C). Sham *Mttp-IKO* mice exhibited significantly longer villi and deeper crypts compared to control shams, as previously observed [29]. In line with these morphologic changes, pneumonia decreased intestinal proliferation in control mice as measured by BrdU labeling of S-phase cells (Figure 2D). In contrast, septic *Mttp-IKO* mice exhibited a normalized proliferative response and similar proliferative capacity as sham *Mttp-IKO* mice, both groups exhibiting increased proliferation compared to control shams. The defects in chylomicron assembly in *Mttp-IKO* mice were accompanied by the presence of large intracellular lipid droplets (Figure 2E) and a corresponding increase in mucosal triglyceride content (Figure 2F). However, there was no change in mucosal triglyceride abundance in control or septic sham mice and mucosal cholesterol content was comparable in all four groups of mice (Figure 2G). These data together suggest that there is a relatively specific impairment of triglyceride mobilization from the intestine of *Mttp-IKO* mice as a result of defective chylomicron assembly.

### Pneumonia-induced Intestinal Epithelial Apoptosis is Prevented in Mice with Impaired Intestinal Lipid Transport

**Figure 3:**
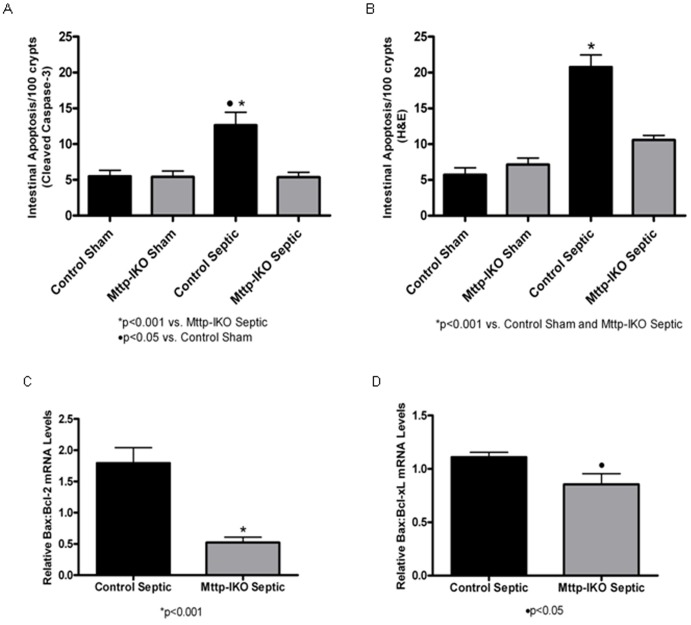
Effect of impaired intestinal lipid transport on intestinal epithelial apoptosis. Intestinal epithelial apoptosis was evaluated by active caspase-3 staining (A) and H&E staining (B) in 100 crypts. Control mice subjected to *P. aeruginosa* pneumonia exhibited increased intestinal apoptosis by both methods. In contrast, *Mttp-IKO* mice with pneumonia had similar levels of intestinal apoptosis as sham mice.n = 6–7 shams/genotype, n = 16–18 septics/genotype. The gene expression ratios of pro-apoptotic Bax to anti-apoptotic Bcl-2 (C) and Bcl-xL (D) were evaluated. Septic *Mttp-IKO* mice had significantly decreased ratios compared to septic control mice. n = 11/group.

Intestinal epithelial apoptosis was increased in control mice subjected to pneumonia compared to sham mice, both when assayed by active caspase-3 staining (Figure 3A) and also by morphological criteria in H&E-stained sections (Figure 3B). In contrast, septic *Mttp-IKO* mice exhibited normalization of apoptosis, to levels observed in sham mice. The ratio of pro-apoptotic to anti-apoptotic molecules is often used as an indicator of sensitivity to apoptosis [29], [30]. Septic *Mttp-IKO* mice exhibited significantly decreased mRNA abundance ratios of both Bax/Bcl-2 (Figure 3C) and Bax/Bcl-xL (Figure 3D) compared to septic control mice, findings consistent with the morphologic criteria above indicating reduced apoptosis. These findings together suggest that the adverse effects of systemic sepsis on intestinal apoptosis are prevented in mice with defective chylomicron assembly.

We considered the possibility that the changes in intestinal proliferation and apoptosis associated with systemic sepsis might somehow be related to augmented intestinal lipid accumulation. However, this was not the case. Wild-type C57BL/6 mice fed a high fat, cholesterol supplemented diet for 3 weeks prior to induction of *P. aeruginosa* pneumonia exhibited comparable mortality (4/12) at 7 days to mice fed a low-fat chow diet (6/12, p = 0.32). Thus, despite earlier work showing that mice fed a high fat diet exhibit increased mortality from sepsis associated with cecal ligation and puncture [11], our findings indicate that augmenting dietary intestinal lipid alone cannot account for the protection observed against pneumonia-associated sepsis in *Mttp-IKO* mice.

### Parameters of Pulmonary Bacterial Clearance: BAL Cultures and MPO Activity

**Figure 4:**
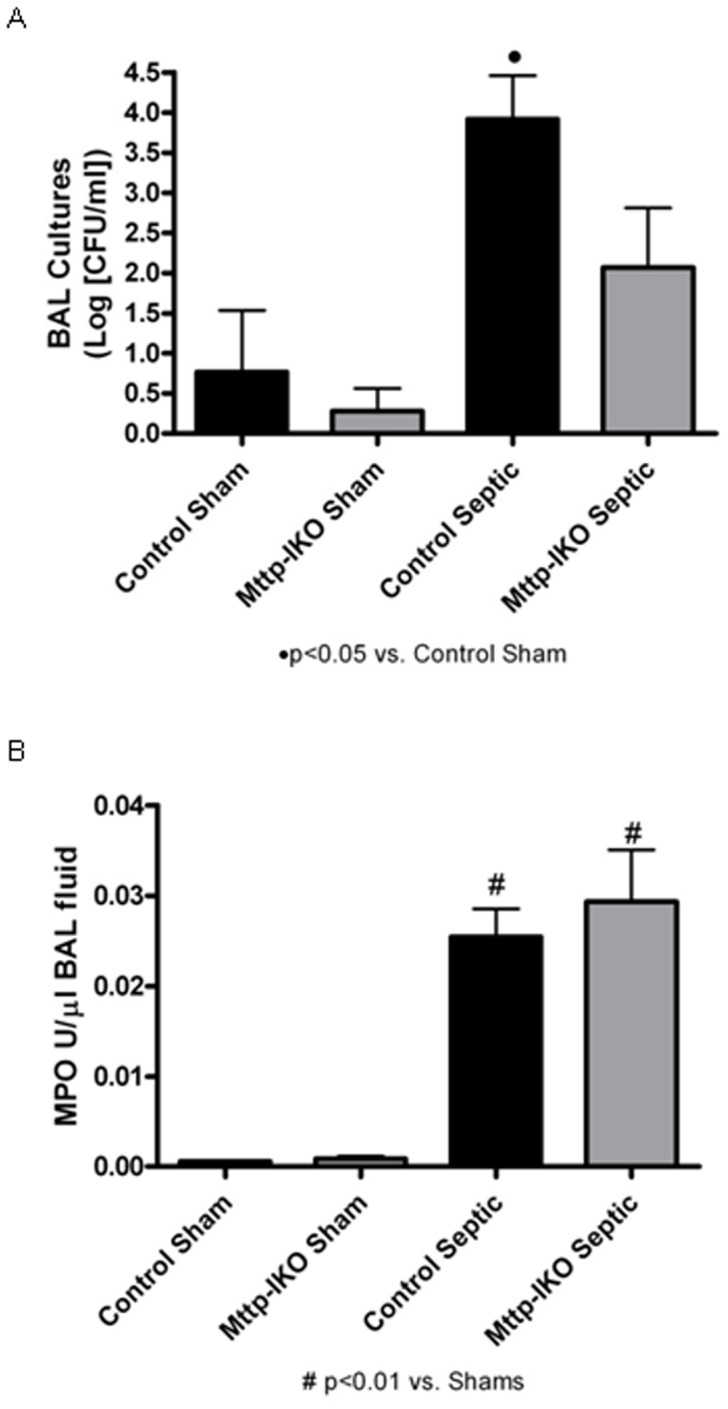
Effect of impaired intestinal lipid transport on pulmonary bacterial clearance and neutrophil infiltration. Control mice with pneumonia had increased bacterial burden in the lungs compared to shams (A). There was less bacteria in the BAL fluid of *Mttp-IKO* mice with pneumonia, but the difference was not statistically significant compared to control septic mice. Myeloperoxidase (MPO) activity was evaluated as an index of neutrophil infiltration and degranulation in BAL fluid (B). Mice subjected to *P. aeruginosa* pneumonia exhibited elevated MPO activity compared to shams; however, the lack of intestinal lipid absorption did not significantly alter neutrophil activation. n = 3–5 shams/genotype, n = 8–10 septics/genotype.

We next turned our attention to the effects of impaired intestinal lipid transport on pulmonary inflammation, to determine if the attenuated mortality in *Mttp-IKO* mice reflected improvements in pulmonary bacterial clearance. Control septic mice (as expected) exhibited significantly increased bacterial burden in the lungs compared to shams, as indicated by the increased BAL culture yield (Figure 4A). There was a trend to reduced bacterial content in the BAL fluid of *Mttp-IKO* mice with pneumonia, but the difference was not statistically significant compared to control septic mice. Both control and *Mttp-IKO* septic mice exhibited elevated MPO activity in BAL fluid, with no differences by genotype between the two septic groups (Figure 4B). These findings together suggest that the improvement in survival in *Mttp-IKO* septic mice was not the result of alterations in pulmonary bacterial clearance as inferred from the culture results and MPO activities in BAL fluid. It is possible that determinations on whole lung parenchyma might reveal additional differences but this was not undertaken.

### The Effect of Impaired Intestinal Lipid Transport on Cytokine Levels

**Figure 5:**
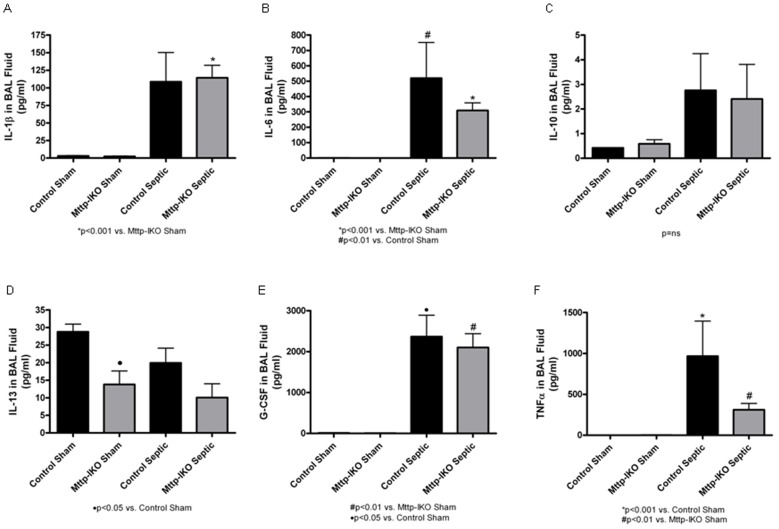
Effect of impaired intestinal lipid transport on lung cytokines. Cytokines were measured in bronchoalveolar lavage (BAL) fluid 24 hr after induction of pneumonia. Although several cytokines were elevated during pneumonia, there were no differences between septic *Mttp-IKO* and control mice. n = 8–10/group.

We next examined whether impaired chylomicron formation altered the local (ie BAL) and systemic inflammatory response to *P. aeruginosa* pneumonia-induced sepsis. The levels of several pro- and anti-inflammatory cytokines (including IL-1β, IL-6, IL-10, IL-13, G-CSF and TNFα) were examined in BAL fluid 24 hours after the onset of pneumonia in both genotypes (Figure 5A–F). However, while there were statistically significant increases accompanying sepsis in IL-6 and G-CSF and trends to higher levels with IL-1β and IL-10, there were no significant differences in the levels of these cytokines in comparing control and *Mttp-IKO* septic mice. The levels of TNFα were also increased in sepsis, with a trend to lower levels in *Mttp-IKO* septic mice (Figure 5F). By contrast, the levels of IL-13 in BAL fluid tended to decrease in septic animals.

**Figure 6:**
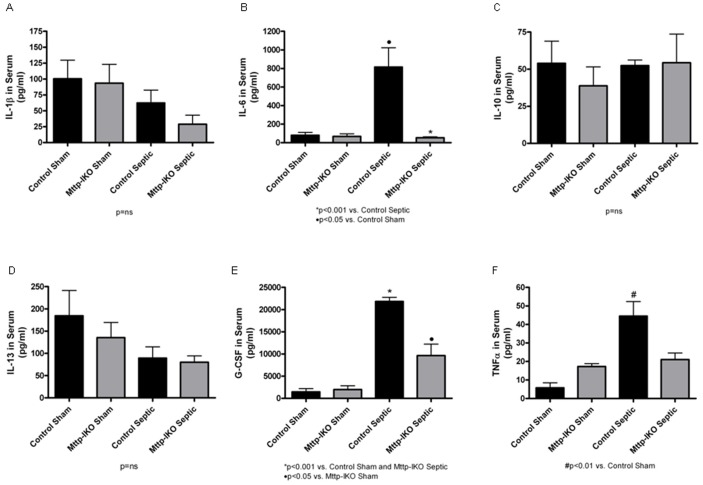
Effect of impaired intestinal lipid transport on systemic cytokines. Cytokines were measured in serum 24 hr after induction of pneumonia. The proinflammatory cytokines IL-6 and G-CSF were increased in septic control mice; however, these cytokines were reduced in septic *Mttp-IKO* mice. n = 4–5 shams/genotype, n = 13–15 septics/genotype.

We also examined serum levels of these same pro- and anti-inflammatory cytokines (Figure 6A–F), which revealed important differences from the patterns observed above. Among the most striking findings was the ∼15-fold increased serum levels of IL-6 in septic control mice (816±208 pg/ml) compared to septic *Mttp-IKO* mice (54±10 pg/ml) and significant differences in serum levels of G-CSF septic control mice (21835±945 pg/ml) compared to septic *Mttp-IKO* mice (9636±2575 pg/ml). No statistically significant differences were observed in serum levels of IL-13 or IL-10, or IL-1ß following pneumonia. The observation that BAL levels of IL-6 were increased in septic animals of both genotypes while serum levels of IL-6 were elevated only in septic control mice raises the important question of how intestinal Mttp deletion might influence serum IL-6 kinetics in the setting of sepsis.

**Figure 7:**
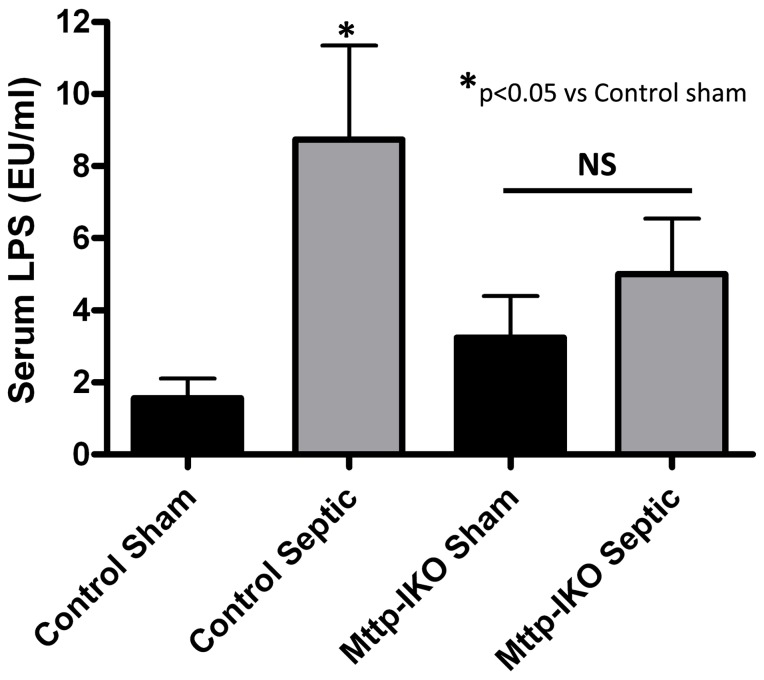
Effect of impaired intestinal lipid transport on serum lipopolysaccharide (LPS). Serum LPS was increased in septic control mice, but not in septic *Mttp-IKO* mice. Serum LPS concentration was measured using the LAL chromogenic endotoxin kit (Methods). N = 5–8 per group. *p<0.05.

We addressed this question indirectly by demonstrating that serum LPS levels were significantly increased in septic control but not septic *Mttp-IKO* mice (Figure 7A), findings consistent with the observation that chylomicron secretion tends to promote the intestinal production of LPS [31]. In light of recent findings demonstrating that LPS augments production of IL-6 in CD25+ regulatory T cell/mast cell cocultures and regulates intestinal IL-6 production [32], these findings support the hypothesis that blocking chylomicron secretion (and decreasing LPS transport) attenuates the release of mediators of systemic sepsis (including IL-6), the latter arising most likely from stromal cells of the small intestine.

### Alterations in Serum Lipoprotein Distribution in Sepsis

**Figure 8:**
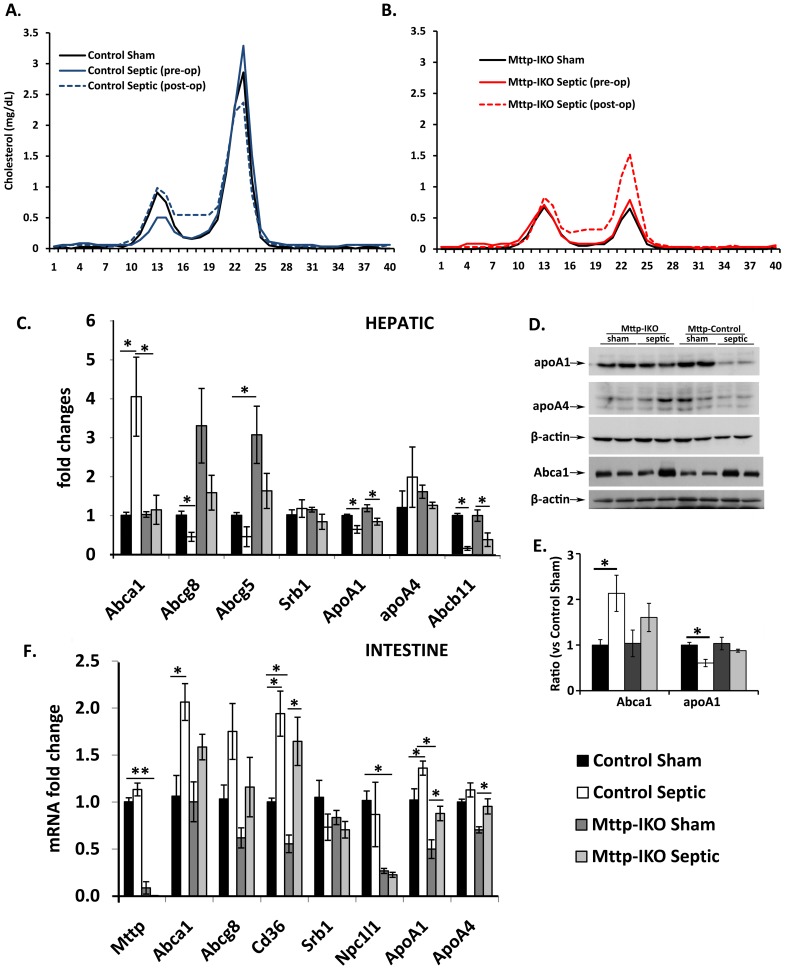
Effect of impaired intestinal lipid transport on serum lipoproteins. Lipoprotein distribution measured by fast protein liquid chromatography in control (A) and *Mttp-IKO* (B) mice. Pooled samples of serum from n = 5–10 mice per genotype were analyzed in sham animals and both before and 24 hr after induction of pneumonia in the experimental groups. Cholesterol was assayed enzymatically and peaks corresponding to fractions 10–16 indicate particles in the low density lipoprotein (LDL) range while fractions 19–26 correspond to high density lipoprotein (HDL). (C) Expression of genes implicated in hepatic cholesterol efflux were analyzed by qRT-PCR on samples of RNA from the indicated groups (n = 4 mice per genotype and treatment) (D) and (E). Expression of hepatic Abca1, apoA1 and apoA4 protein by SDS-PAGE and western blot. Gapdh was used as loading control. Panel D shows representative Western blotting results. Panel E shows densitometric scanning from groups of 4 mice per genotype and treatment. (F) Expression of genes implicated in intestinal lipid metabolism were analyzed by qRT-PCR on samples of small intestinal RNA from the indicated groups (n = 4 mice per genotype and treatment). *p<0.05.

Pneumonia did not alter the total serum concentrations of cholesterol, triglycerides, phospholipids, or free fatty acids in control mice (Table 1). However, when examining the individual lipoprotein distribution of cholesterol, there was a subtle but consistent decrease in HDL cholesterol in the septic control mice (Figure 8A). By contrast, septic *Mttp-IKO* mice exhibited significantly increased serum levels of cholesterol and phospholipids compared to sham *Mttp-IKO* mice (Table 1), along with a striking increase in HDL cholesterol concentrations (Figure 8B). Serum LDL cholesterol levels were generally decreased in *Mttp-IKO* mice compared to control mice, regardless of the presence of pneumonia (Figure 8B).

**Table 1:** Impaired intestinal lipid transport alters serum lipid concentrations in septic mice.

	TG (mg/dL) (pre-op)	TG (mg/dL) (post-op)	Chol (mg/dL) (pre-op)	Chol (mg/dL) (post-op)	PL (mg/dL) (pre-op)	PL (mg/dL) (post-op)	FFA (mmol/L)(pre-op)	FFA (mmol/L)(post-op)
**Control Sham**	90.5±9.7	53.5±5.9	78.8±7.5	91.4±5.8	188.7±25.6	177.7±12.5	0.53±0.2	0.29±0.1
***Mttp-IKO*** ** Sham**	77.0±13.0	52.2±8.5	35.3±4.0^a^	42.8±6.6^b^	80.4±5.9^a^	85.1±9.3^b^	0.47±0.1	0.43±0.1
**Control Septic**	62.1±12.0	31.2±8.5	84.6±6.9	95.3±6.0	181.1±14.8	154.6±11.1	0.51±0.1	0.54±0.1
***Mttp-IKO*** ** Septic**	56.1±6.5	29.2±4.0	44.5±2.7^e^	88.5±8.2c,d	78.6±4.1^e^	149.0±13.9d,f	0.34±0.1	0.37±0.1

TG = triglycerides, Chol = cholesterol, PL = phospholipid, FFA = free fatty acids.

All results are expressed as mean ± SEM.

a p<0.001 vs. Pre Control Sham;

b p<0.001 vs. Post Control Sham;

c p<0.001 vs. Post Control Septic;

d p<0.001 vs. Pre *Mttp-IKO* Septic;

e p<0.001 vs. Pre Control Septic;

f p<0.01 vs. Post *Mttp-IKO* Sham.

To begin to understand the mechanisms for the increased HDL levels in septic *Mttp-IKO* mice, we examined a panel of candidate genes implicated in cholesterol efflux from both the liver and small intestine. This revealed significantly increased mRNA abundance of the basolateral cholesterol efflux pump Abca1in the liver, and intestine of septic control mice (Figure 8C, F). A correspondingly increased expression of hepatic Abca1 protein was also demonstrated (Figure 8D, E). To reconcile how serum HDL levels might be decreased in the face of increased expression of Abca1, a finding previously implicated in promoting HDL biogenesis [33], [34], we also examined hepatic and intestinal expression of apolipoprotein A-1 (apoA1), the dominant protein component of circulating HDL. Those studies revealed decreased hepatic apoA1 mRNA in septic animals (Figure 8C), coupled with a marked decrease in hepatic apoA1 protein in septic control but not septic *Mttp-IKO* mice (Figure 8D). These findings imply that both transcriptional and post-transcriptional mechanisms may account for the decrease in hepatic apoA1 expression in septic control mice and may at least partially account for the reduced HDL levels observed in the setting of increased Abca1 expression. On the other hand, despite a small, albeit significant decrease in hepatic apoA1 mRNA abundance in septic *Mttp-IKO* mice, expression of hepatic apoA1 protein was preserved in these animals (Figure 8D). Intestinal apoA1 and apoA4 mRNA abundance was increased in septic animals of both genotypes (Figure 8E), but protein expression could not be unequivocally determined because of variable degradation in mucosal extracts (data not shown). Taken together, the findings suggest that HDL biogenesis is impaired in the setting of sepsis through a combination of mechanisms that include decreased hepatic apoA1 expression.

Sepsis was also associated with decreased mRNA abundance of hepatic Abcg5/g8 expression (the canalicular cholesterol transporter) and down regulation of the bile acid transporter Abcb11 (Figure 8C). Those findings are in accord with other studies that suggest that inflammation impairs the pathways of reverse cholesterol transport and biliary excretion in-vivo [35]. The decreases were qualitatively similar in septic mice of both genotypes, although the expression of hepatic Abcg5/g8 tended to be greater in *Mttp-IKO* mice, as recently demonstrated [28]. In addition, sepsis was associated with increased mRNA abundance of the apical fatty acid transporter Cd36 with comparable induction in both genotypes (Figure 8E). Expression of other cholesterol transporters, including Srb1, Npc1L1 were unchanged in both the liver and intestine of both genotypes and there was no change in intestinal Mttp expression in septic control mice (Figure 8C, E). The findings together reveal a complex network of adaptive pathways in both intestinal and hepatic lipid metabolism in sepsis, some of which are selectively attenuated (hepatic Apo A1 expression) in the setting of intestinal Mttp deletion. In particular, the findings reinforce the concept that deletion of intestinal Mttp results in a cascade of adaptive changes in hepatic lipid metabolism that may contribute to the overall protective effects observed in sepsis [28].

## Discussion

This study demonstrates that defective intestinal chylomicron assembly in *Mttp-IKO* mice confers a survival advantage in mice subjected to *P. aeruginosa* pneumonia. This survival advantage is associated with decreased pneumonia-induced intestinal atrophy and epithelial apoptosis, preservation of the intestinal proliferative response, and a reduction in circulating levels of the proinflammatory cytokines IL-6 and G-CSF. These changes occurred without significant alterations in bacterial clearance from the lungs, pulmonary neutrophil infiltration, or pulmonary cytokine levels in septic *Mttp-IKO* compared to control septic mice. Several features of these core observations merit further discussion.

Critically ill patients frequently manifest decreased serum lipid levels and may become hypolipoproteinemic [36]. However, the functional significance of this observation is unclear since on the one hand lipoprotein infusion is protective against lethality in endotoxemia [37], while on the other hand, hyperlipoproteinemic LDL receptor-deficient mice manifest increased mortality following cecal ligation and puncture [38]. Lipoproteins are also known to be important regulators of the immune response due to their ability to neutralize lipopolysaccharide (LPS) [39], [40]. In addition, intestinal absorption of dietary fat from the enterocyte into the mesenteric lymph via chylomicrons also facilitates the absorption of bacterial LPS from the intestinal lumen and promotes delivery to regional mesenteric lymph nodes [31]. In this scenario, it is feasible that sequestration of absorbed LPS on chylomicrons would reduce endotoxin toxicity, as inferred from studies in isolated hepatocytes [41]. However, it is also possible that chylomicrons mediate systemic pro-inflammatory effects that correlate with LPS content. Indeed, Ghoshal et al. found that intestinal LPS absorption was dependent on chylomicron formation in mice, since administration of Pluronic L-81, an inhibitor of chylomicron secretion, significantly decreased blood LPS levels [31]. Our findings are consistent with these latter data and demonstrate a significant protective effect against pneumonia-induced sepsis in *Mttp-IKO* mice, where chylomicron assembly is virtually eliminated. In particular, the finding that serum LPS levels were not significantly different in septic and sham *Mttp-IKO* mice (Figure 7) supports the suggestion that blocking intestinal chylomicron secretion also attenuates the release of LPS from enterocytes in the setting of systemic sepsis. The mechanisms underlying the observed reduction in serum IL-6, however, are unlikely to reflect enterocyte secretion, since the source of intestinal IL-6 is believed to be subepithelial lamina propria myofibroblasts [42], although the precise sources(of serum IL-6) were not formally explored in the current studies and we did not perform studies to examine IL-6 clearance.

The demonstration that survival in pneumonia-induced sepsis in *Mttp-IKO* mice was associated with increased concentrations of HDL is intriguing in light of findings in patients with severe sepsis where a rapid decline in HDL levels has been observed [39]. One suggestion emerging from our findings in septic *Mttp-IKO* mice is that strategies to raise HDL levels may be protective. This suggestion is consistent with other findings that injection of an apolipoprotein A1 mimetic (the major protein on HDL) peptide attenuates sepsis in rats [43]. More recent work has extended these findings by demonstrating that HDL inhibits a subset of lipopolysaccharide induced macrophage genes regulating the type 1 interferon response [44]. Our findings suggest that serum HDL production may be selectively increased in septic *Mttp-IKO* mice as inferred by a combination of increased intestinal and hepatic Abca1 expression, retained expression of hepatic apoA1 protein and increased intestinal apoA1 mRNA expression. The findings are consistent with other studies that demonstrate impaired reverse cholesterol transport and HDL-mediated cholesterol delivery to bile in septic control mice [35]. It is plausible that other compensatory changes, for example increased HDL turnover in control septic mice, may account for some of the changes observed and this possibility is worth consideration in future studies.

It has been well established in models of hemorrhagic shock that toxic, gut-derived lipid factors are carried in the mesenteric lymph to the systemic circulation where they secondarily induce distant organ injury [7]. Importantly, lung injury induced by shock can be completely abrogated by ligation of the mesenteric lymph duct in animal models, again implying a key role for intestinally derived mediators [7], [9]. However, although gut-derived factors responsible for injury in hemorrhagic shock have been found in the lipid fraction of lymph, their exact identity remains unknown and the lipoprotein carriers have not been fully defined [45]. In addition, the current findings demonstrate that defective chylomicron assembly and the corresponding decrease in lipid transport into mesenteric lymph improved the overall outcome but did not actually prevent lung injury. Taken together, our observations suggest that eliminating intestinal chylomicron secretion in the setting of sepsis leads to reduced systemic inflammation that in turn is associated with attenuation of intestinal injury. This conclusion is broadly consistent with other findings that support a role for intestinally absorbed antigens in promoting mesenteric adipose inflammation in mice during high fat feeding [46]. As alluded to above, we explored the possibility that exposing wild-type control mice to a high fat high cholesterol diet might influence the response to pneumonia associated sepsis, but our results indicated no such effect. However, the effects of prolonged high fat feeding and altered intestinal lipid flux should be considered in light of the associated effects of obesity and the altered profile of adipokines that modulate innate immunity. For example, studies have demonstrated that leptin administration corrects impaired host defense pathways in the setting of mice starved for 48 h prior to induction of pneumococcal pneumonia [47], [48]. Other studies have demonstrated that short term high fat feeding (3 weeks) increases organ injury and mortality associated with cecal ligation and puncture, suggesting that some models of sepsis are indeed responsive to dietary fat intake [11]. We did not examine this model of polymicrobial sepsis in *Mttp-IKO* mice but this approach might be considered in future studies.

Although pneumonia originates within bronchoalveolar tissues, these infections may lead to severe extrapulmonary consequences, often of equal if not greater, physiologic significance. In relation to the current findings, it is known that mice with *P. aeruginosa* pneumonia-induced sepsis exhibit increased intestinal epithelial apoptosis and decreased proliferation [17], [49].In the current study, apoptotic cells were identified primarily in the crypts, leading us to speculate that depletion of stem cells or proliferating daughter cells may prevent renewal of the crypt-villus structure and eventually causing mucosal atrophy. These defects in gut homeostasis may be self-sustaining and ultimately lead to perpetuation of the systemic inflammatory response. We found that septic *Mttp-IKO* mice maintained the adaptive changes in intestinal proliferation and decreased apoptosis, the net result reflecting a normalized villus/crypt architecture. The precise mechanisms by which impaired chylomicron formation results in these adaptive changes in intestinal proliferation and apoptosis are under active investigation. The precise pathways involved are unknown but based on preliminary unpublished data, we speculate that altered production of incretins may be involved.

Several features of altered intestinal lipid metabolism in *Mttp-IKO* mice recapitulate features in human subjects with abetalipoproteinemia. These include intestinal lipid malabsorption and reduced circulating levels of serum lipids [23]. However, patients with abetalipoproteinemia also manifest hepatic steatosis because they also lack MTTP in hepatocytes. Our observations in *Mttp-IKO* mice raise the question of whether a similar protective effect would be observed in mice with conditional, liver-specific Mttp deletion [50]. Further study will be required to answer this question definitively. Pharmacologic compounds that block Mttp activity are currently in clinical trials as lipid lowering agents, but these drugs have the drawback of inhibiting both hepatic and intestinal Mttp [51]. Nevertheless, the findings raise the intriguing possibility that pharmacologic agents that block intestinal chylomicron secretion selectively (in other words an intestine-specific Mttp inhibitor [52]) might offer a possible therapeutic for subjects with systemic sepsis.

Although the current study provides important insights into the role of gut-derived lipids in the progression of pneumonia-induced sepsis, we acknowledge a number of limitations. While no major differences were found in pulmonary bacterial clearance between *Mttp-IKO* and control septic mice, this does not conclusively preclude the possibility that differences exist since our assay design was limited to 24 hr after the onset of pneumonia and later time points were not examined. In addition, there may be other mediators, in both the lung and intestine, through which conditional ablation of intestinal lipid transport prevents sepsis-induced intestinal injury. Despite these limitations, this study demonstrates that decreasing intestinal lipid transport by preventing chylomicron assembly and secretion prevents intestinal injury and improves survival in *P. aeruginosa* pneumonia. The findings reinforce the concept that gut-derived lipids play an important role in the progression of sepsis, as seen in models of shock and ischemia-reperfusion injury.
